# Factors Associated With Edoxaban Concentration Among Patients With Atrial Fibrillation

**DOI:** 10.3389/fphar.2021.736826

**Published:** 2021-09-09

**Authors:** Shin-Yi Lin, Ching-Hua Kuo, Li-Ting Ho, Yen-Bin Liu, Chih-Fen Huang, Sung-Chun Tang, Jiann-Shing Jeng

**Affiliations:** ^1^ Department of Pharmacy, National Taiwan University Hospital, Taipei, Taiwan; ^2^ School of Pharmacy, College of Medicine, National Taiwan University, Taipei, Taiwan; ^3^ Cardiovascular Center and Division of Cardiology, Department of Internal Medicine, National Taiwan University Hospital, Taipei, Taiwan; ^4^ Stroke Center and Department of Neurology, National Taiwan University Hospital, Taipei, Taiwan

**Keywords:** atrial fibrillation, edoxaban, factor Xa inhibitors, therapeutic drug monitoring, Asians

## Abstract

**Background and Purpose:** Edoxaban exposure varies across different ethnicities. The purpose of our study was to examine the risk factors associated with high or low edoxaban concentrations in Asian populations.

**Methods:** Participants with atrial fibrillation who were undergoing edoxaban therapy were enrolled. Peak (1–4 h after edoxaban administration) and trough (24 ± 4 h from the last edoxaban dose) blood samples were collected to measure edoxaban concentrations using ultrahigh-performance liquid chromatography with tandem mass spectrometry. The edoxaban concentrations were compared to those observed in clinical trials to define a higher- or lower-than-expected range. Multivariate logistic regression was used to analyze the risk factors associated with high or low edoxaban concentrations.

**Results:** Eighty participants (49 men, 61.3%) were enrolled and provided 78 trough and 76 peak samples. Twenty participants (25.6%) were determined to have low trough concentrations, which was associated with higher creatinine clearance and the use of the 30 mg regimen (odds ratio [OR] and 95% confidence interval [CI], 1.06 [1.01, 1.11], *p* = 0.01 and 5.77 [1.34, 24.75], *p* = 0.02, respectively). In contrast, 21 participants (27.6%) had high peak concentrations, which was associated with an off-label overdosing regimen (OR = 4.68 [1.23, 17.70], *p* = 0.02).

**Conclusion:** Our study identified factors associated with increased or decreased edoxaban exposure. The measurement of edoxaban concentration may be recommended for patients with selected characteristics.

## Introduction

Edoxaban is a direct, reversible inhibitor of factor Xa that is administered once daily (Parasrampuria and Truitt, 2016; Steffel et al., 2021). It displays similar efficacy in preventing thromboembolism among patients with atrial fibrillation (AF) while reducing the risk of bleeding in comparison to warfarin. In addition, edoxaban reduces cardiovascular mortality (Giugliano et al., 2013). Edoxaban exhibits linear pharmacokinetics. Exposure to edoxaban is proportional to the dose (Parasrampuria and Truitt, 2016). The bioavailability of edoxaban is approximately 62%, and its absorption is minimally affected by food. After administration, the plasma concentration peaks after 1–2 h, and the half-life ranges from 10 to 14 h (Steffel et al., 2021). Approximately 50% of edoxaban is cleared in the urine in an unchanged form, and the other 50% undergoes nonrenal clearance, which includes biliary secretion of an unchanged drug and hepatic metabolism, such as metabolism by carboxylesterase-1 (CES1), cytochrome P3A4/P3A5, enzyme hydrolysis, and glucuronidation. (Parasrampuria and Truitt, 2016). Approximately 10% of total edoxaban undergoes hepatic metabolism (Parasrampuria and Truitt, 2016). The standard dose of edoxaban administered to patients with AF is 60 mg daily. In patients with impaired renal function, low body weight, and concomitant use of medications that potently inhibit P-glycoprotein (P-gp), the dose should be reduced to 30 mg daily (de Vries et al., 2013; Steffel et al., 2021). In the Edoxaban Low-Dose for Elder Care Atrial Fibrillation Patients (ELDERCARE-AF) trial conducted in very elderly Japanese patients, a very-low-dose regimen (15 mg daily) was shown to be effective in preventing stroke and systemic embolism and did not increase the risk of bleeding compared with the placebo. (Okumura et al., 2020).

In the subanalysis of the Effective Anticoagulation with Factor Xa Next Generation in Atrial Fibrillation-Thrombolysis in Myocardial Infarction 48 (ENGAGE AF-TIMI 48) trial, the edoxaban concentration was consistently lower in Asians than in non-Asians, regardless of whether the edoxaban dose was adjusted (Chao et al., 2019). More importantly, the increase in the risk of major bleeding and intracranial hemorrhage (ICH) as the edoxaban concentration increased was steeper in Asians than in non-Asians. A specific cutoff value for the increased ischemic stroke and ICH risk has been proposed for Asians but not for non-Asians due to the persistently low risk of bleeding. (Chao et al., 2019). As both the exposure and response to edoxaban vary across different ethnicities, (Chao et al., 2019) we established a cohort of edoxaban therapies and collected data on edoxaban concentrations. Thus, this study aims to investigate the potential risk factors associated with high or low concentrations of edoxaban.

## Methods

### Study Design

This prospective observational study was conducted at the National Taiwan University Hospital (NTUH) from December 01, 2018, to March 31, 2020. The study protocol was approved by the International Ethics Committee of the NTUH. Each participant provided written informed consent before enrolling in this study. Participants over 20 years of age who had AF and were treated with edoxaban therapy for more than 7 days met the inclusion criteria. Patients who were pregnant or breastfeeding, who failed to provide at least one blood sample, or who declined to provide written informed consent were excluded from this study.

### Measurement of the Plasma Edoxaban Concentration

Two blood samples were collected through venous puncture and stored in tubes containing K2EDTA (BD Vacutainer^®^). Participants were asked to refrain from taking the scheduled morning dose of edoxaban before the collection of the trough sample, which was 24 ± 4 h from the last edoxaban dose. The edoxaban administration time on the day before the study date was self-reported by the participants and was used to determine the time that had elapsed from the last edoxaban dose. After the trough sample was collected, participants were asked to take an edoxaban dose under the supervision of a pharmacist and then stay in the hospital until peak sample collection, which occurred 1–4 h after edoxaban administration.

Blood samples were centrifuged using a standard procedure to obtain the plasma, which was then stored in a −80°C freezer. The edoxaban concentration was then analyzed using ultrahigh-performance liquid chromatography with tandem mass spectrometry. A detailed description of the analytical method is provided in Supplementary Text S1.

Briefly, an Agilent 1290 UHPLC system coupled with a mass spectrometer (Agilent 6460 triple quadrupole system [Agilent Technologies, Waldbronn, Germany]) and a Kinetex reverse-phase core-shell C18 column (2.1 × 50 mm, 2.6 μm, 100 Å, Phenomenex, Torrance, CA, United States) were used for the concentration analysis. The mobile phase consisted of 0.1% formic acid and 10 mM ammonium acetate in water for solvent A and 0.1% formic acid and 10 mM ammonium acetate in isopropanol and ACN (9:1, v/v) for solvent B. The flow rate was 0.35 ml min^−1^. The gradient profile started with 0% B for 0.5 min. Then, it changed to 6% B in 0.1 min and remained at 6% B for 0.6 min. The gradient profile was subsequently increased to 25% B in 0.5 min, 27.5% B in 0.5 min, 50% B in 0.5 min, and was maintained at 50% B for 1 min. Finally, the column was re-equilibrated to 0% B for 2 min until the next injection. The temperature of the sample reservoir was maintained at 4°C, and the column oven temperature was set to 55°C. The injection volume was 3 μl. The positive electrospray ionization mode was utilized with the following parameters: 350°C dry gas temperature, 10 L min^−1^ dry gas flow rate, 45 psi nebulizer pressure, 350°C sheath gas temperature, 11 L min^−1^ sheath gas flow rate, 3,500 V capillary voltage, and 500 V nozzle voltage. MS acquisition was executed in multiple reaction monitoring (MRM) mode. Two transitions were selected as a quantifier and qualifier: 548.1→ 366.1 and 548.1 → 152 for edoxaban and 554.1 → 372.1 and 554.1 → 158.1 for [*d*_6_]-edoxaban, respectively.

### Clinical Data Acquisition

The following data were prospectively recorded from the electrical medical records: (1): demographic characteristics (age and sex), (2), comorbidity conditions (ischemic stroke, transient ischemic attack [TIA], congestive heart failure, hypertension, diabetes, myocardial infarction, peripheral arterial occlusive disease, malignancy, and bleeding history), (3), order details and concurrent medications (dose; frequency; starting and end date of edoxaban prescription; concurrent use of P-gp inducers or inhibitors, including amiodarone, dronedarone, verapamil, quinidine, rifampin, azole antifungal agents, macrolide antibiotics, anti-retroviral drugs, antiepileptic drugs; and concurrent use of platelet inhibitors, including aspirin, clopidogrel, ticagrelor, prasugrel, other antiplatelet agents, and nonsteroidal anti-inflammatory drugs), and (4) laboratory data (renal and liver function tests and complete blood count). The risk of thromboembolism was estimated using the CHADS_2_-VASc score, (Lip et al., 2010), and the risk of bleeding was estimated using the HAS-BLED score (Pisters et al., 2010). The item labile INR was not included in this study because most of the participants were newly treated with non-vitamin K antagonist oral anticoagulants (NOAC) or shifted from other NOAC, rather than warfarin, before using edoxaban.

Based on the ELDERCARE-AF trial, we also classified all the participants into fragile and nonfragile groups and compared the differences in edoxaban concentrations (Okumura et al., 2020). Participants aged ≥80 years with a CHADS2 score of ≥2 points (Gage et al., 2001) and any one of the following features were classified into the fragile group: weight ≤45 kg, creatinine clearance (CrCL) within 15–30 ml/min, major or gastrointestinal bleeding history, and concurrent use of nonsteroidal anti-inflammatory drugs or antiplatelet agents.

### Adherence

Edoxaban adherence was measured using a self-report questionnaire. An investigator recorded whether the edoxaban dose was skipped for 7 days before blood concentration monitoring. The frequency of a missed dose of edoxaban during treatment was also recorded, along with the reason the dose was skipped. Good adherence was defined as no missed dose 1 week before blood sample collection and seldom skipped edoxaban dose during treatment (≤1 time per month). We then analyzed the effect of edoxaban adherence on drug concentrations.

### Definitions of On-Label and Off-Label Dosing Regimens

Participants were stratified into on-label dosing, off-label underdosing and off-label overdosing groups to compare the differences in edoxaban concentrations. The definitions for on- or off-label dosing regimens were determined according to the package insert (Granger et al., 2011; Giugliano et al., 2013; Hindricks et al., 2020). The standard dose used to prevent AF-associated thromboembolism was 60 mg daily. Patients who met one of the following criteria should have received a reduced dose of edoxaban of 30 mg daily: body weight ≤60 kg, CrCL <50 ml/min, and concurrent use of potent P-gp inhibitors, such as cyclosporin, erythromycin, dronedarone and ketoconazole.

### Factors Associated With Out-Of-Expected Range Edoxaban Concentration

The study endpoint was factors influencing lower-than-expected-range trough concentration and higher-than-expected-range peak concentrations. We focused specifically on out-of-expected-range concentration because the associations between low concentration and increased risk of ischemic stroke and high concentration and increased risk of major bleeding have been reported in clinical trials (Chao et al., 2019). To define the lower-, within- or higher-than-expected-range edoxaban concentration, the edoxaban concentrations were compared with the expected range reported in clinical trials, which was 12–43 ng/ml for the trough concentration and 101–288 ng/ml for the peak concentration (Ogata et al., 2010; Cuker and Husseinzadeh, 2015; Steffel et al., 2021). To investigate factors associated with lower-than-expected-range trough concentrations, participants with and without lower trough concentrations were compared. To the opposite, to investigate factors associated with higher-than-expected range peak concentrations, participants with and without high peak concentrations were compared.

### Statistical Analysis

A descriptive analysis was performed to obtain the means, standard deviations, medians, interquartile ranges (IQRs), numbers, and proportions. One-way ANOVA, Kruskal-Wallis H-test or Chi square test was used to compare between groups differences, as appropriate. Post hoc test for pairwise comparison was performed with Scheffe Test to determine which groups were different. Multivariate logistic regression was used to investigate factors associated with lower- or higher-than-expected range edoxaban concentration. In addition to factors which displayed significant difference between groups, we also adjusted age, sex, weight, CrCL and edoxaban dose/appropriateness of edoxaban dose in the model. According to the rule of thumb, every 10 participants is required for adding one covariate to the model in general. Therefore, we determined five pre-specified factors having impact on edoxaban concentration in the multivariate logistic regression analysis should be reasonable. Data were analyzed using IBM SPSS Statistics (version 26.0; IBM Corp., Armonk, NY, United States). The level of statistical significance was set to 0.05.

## Results

### Demographic Characteristics

**FIGURE 1:**
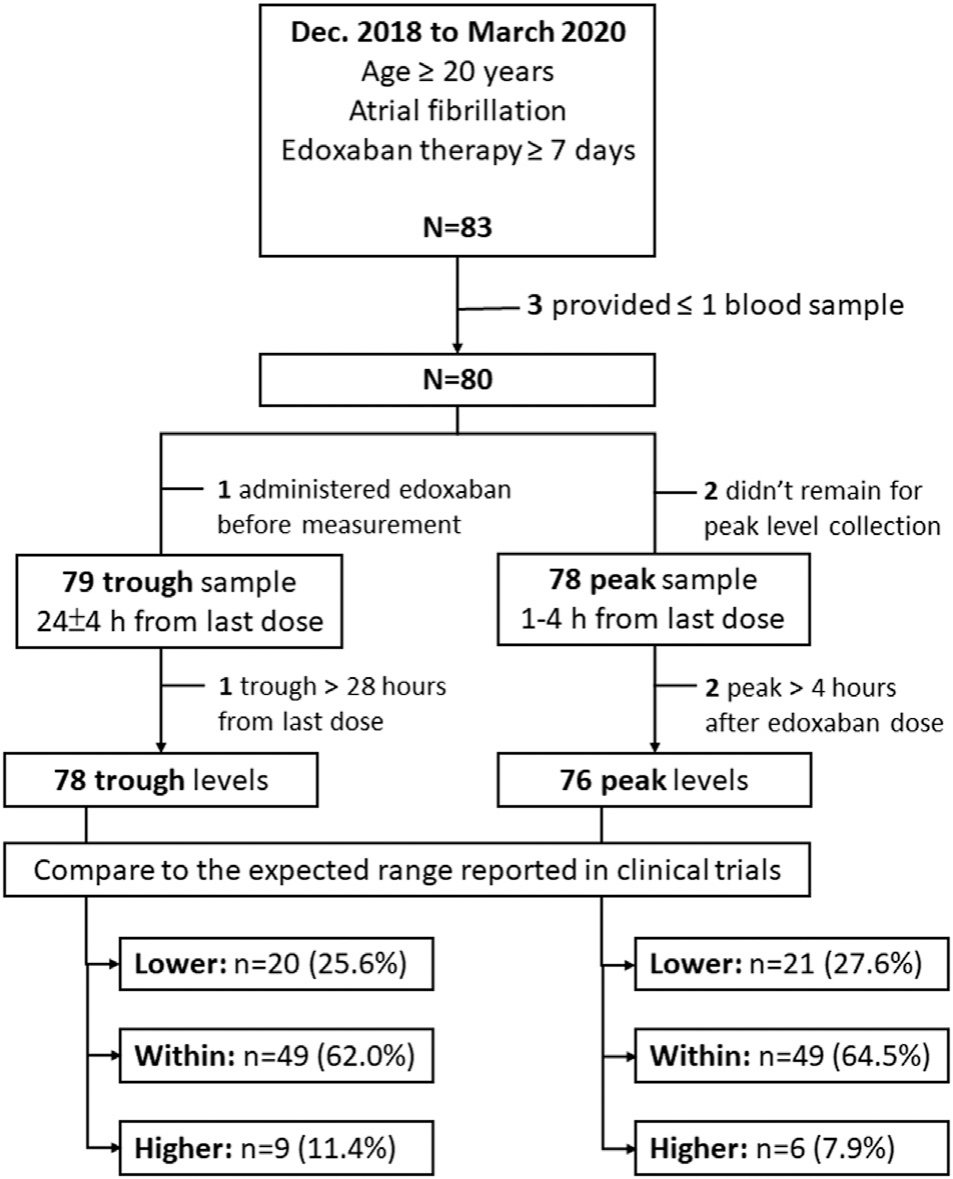
The study enrollment process.

The participant enrollment process is shown in Figure 1. From December 2018 through March 2020, 83 participants were enrolled in this study. Among these participants, 3 were excluded because of a failure to collect at least one edoxaban plasma sample. The remaining 80 participants (49 males, 61.3%) contributed 78 peak and 79 trough samples. After excluding 2 peak and 1 trough samples that were collected after the defined time periods for blood sample collection, 76 peak and 78 trough samples were included for analysis. The mean age of these participants was 74.3 ± 9.7 years, and the mean CHADS_2_-VASc and HAS-BLED scores were 3.9 ± 1.6 and 2.3 ± 1.1 points, respectively. Among all the participants, 43 (53.8%) were prescribed 30 mg daily. The baseline characteristics for all participants were listed in Table 1.

**TABLE 1:** Basic characteristics of participants having within or out-of-expected-range edoxaban trough concentrations.

Characteristic	All participants N = 80	Trough (n = 78, 2 trough samples were missing)		
		Within range n = 49	Lower than range n = 20	Higher than range n = 9
Age (years)	74.3 ± 9.7	73.7 ± 10.4	73.4 ± 8.3	77.8 ± 8.4
Male	49 (61.3)	34 (69.4)	9 (45.0)	6 (66.7)
Weight (kg)	64.9 ± 11.6	66.7 ± 10.6	64.2 ± 12.7	59.8 ± 10.4
BMI (kg/m^2^)	24.7 ± 3.0	25.1 ± 2.6	24.7 ± 3.7	22.4 ± 2.0
CRE (mg/dl)^a^ ^,^ ^b^	1.1 ± 0.5	1.2 ± 0.5	0.9 ± 0.2	1.1 ± 0.2
CrCL	54.9 ± 18.7	53.9 ± 19.0	62.1 ± 15.8	47.4 ± 19.6
ALT (U/L)	24.2 ± 24.1	26.0 ± 26.9	23.3 ± 22.0	15.0 ± 4.8
Comorbidities				
IS or TIA	40 (50.0)	25 (51.0)	7 (35.0)	6 (66.7)
CHF	13 (16.3)	6 (12.2)	3 (15.0)	3 (33.3)
Hypertension	54 (67.5)	35 (71.4)	10 (50.0)	7 (77.8)
Diabetes	15 (18.8)	9 (18.4)	4 (20.0)	1 (11.1)
MI or PAOD	11 (13.8)	6 (12.2)	1 (5.0)	3 (33.3)
Malignancy	15 (18.8)	12 (24.5)	2 (10.0)	1 (11.1)
Dyslipidemia	40 (50.0)	27 (55.1)	9 (45.0)	3 (33.3)
Bleeding history	26 (32.5)	17 (34.7)	4 (20.0)	5 (55.6)
ICH	6 (7.5)	3 (6.1)	0 (0)	2 (22.2)
GI bleeding	6 (7.5)	3 (6.1)	1 (5.0)	1 (11.1)
Other bleeding	17 (21.3)	12 (24.5)	3 (15.0)	2 (22.2)
CHADS2-VASc	3.9 ± 1.6	3.8 ± 1.6	3.4 ± 1.5	4.8 ± 1.6
HAS-BLED^a^	2.3 ± 1.1	2.3 ± 1.1	1.9 ± 0.9	3.0 ± 1.2
Edoxaban concentration (ng/ml)				
Trough^a^ ^,^ ^b^ ^,^ ^c^	24.9 ± 21.9	23.1 ± 8.3	7.8 ± 3.3	72.5 ± 29.5
Peak	239.7 ± 111.0	248.3 ± 110.6	203.6 ± 109.1	290.0 ± 105.6
Edoxaban use				
60 mg daily	37 (46.3)	26 (53.1)	7 (35.0)	4 (44.4)
30 mg daily	43 (53.8)	23 (46.9)	13 (65.0)	5 (55.6)
Off-label overdosing	14 (17.5)	11 (22.4)	2 (10.0)	1 (11.1)
Off-label underdosing	10 (12.5)	5 (10.2)	4 (20.0)	0 (0)
Poor adherence	12 (15.6)	9 (18.8)	2 (10.5)	1 (11.1)
Concurrent medications^d^				
amiodarone	16 (20.0)	12 (24.5)	2 (10.0)	2 (22.2)
dronedarone	2 (2.5)	1 (2.0)	1 (5.0)	0 (0)
verapamil	2 (2.5)	1 (2.0)	0 (0)	0 (0)
NSAID	3 (3.8)	3 (6.1)	0 (0)	0 (0)
Antiplatelet agents^a^	4 (5.0)	2 (4.1)	0 (0)	2 (22.2)

The data are presented as numbers (proportions) or the means ± standard deviations. The 80 participants contributed 78 trough samples. ALT, alanine aminotransferase; BMI, body mass index; CHF, congestive heart failure; CrCL, creatinine clearance; CRE, serum creatinine; GI, gastrointestinal; ICH, intracranial hemorrhage; IS, ischemic stroke; MI, myocardial infarction; N/A, not applicable; NSAID, nonsteroidal anti-inflammatory drug; PAOD, peripheral arterial vascular disease; P-gp, p-glycoprotein; TIA, transient ischemic attack.

a Indicated differences between 3 groups.

b Indicated differences between the low trough and within-range trough groups.

c Indicated differences between the high trough and within-range trough groups.

d Concurrent medications: None of our patients concomitantly used quinidine, azole antifungal agents, protease inhibitors (P-glycoprotein inhibitors), rifampin, or enzyme-inducing antiepileptic drugs such as phenytoin and phenobarbital (P-glycoprotein inducers).

### Edoxaban Concentration

The average trough edoxaban concentration was 24.9 ± 21.9 ng/ml measured from 24.9 ± 1.4 h after the last edoxaban dose. Twenty participants (25.6%) were identified to have lower-than-expected-range trough concentrations and nine participants (11.5%) were determined to have higher-than-expected range trough concentration. The comparisons of 3 trough groups were listed in Table 1. The difference of serum creatinine (CRE) between 3 trough group was significant. Specifically, participants in low trough group had lower CRE level in comparison to within-range group.

The average peak edoxaban concentration was 239.7 ± 111.0 ng/ml, and was measured at 2.0 ± 0.2 h after edoxaban administration. Twenty-one participants (27.6%) had higher-than-expected-range peak concentrations and 6 participants (7.9%) had lower-than-expected peak concentrations. The comparisons of 3 peak groups were listed in Table 2. The differences for proportion of participants with 30 or 60 mg regimen, and with on- or off-label regimen between 3 groups were significant. Specifically, participants in high peak group were more likely to use 60 mg regimen and off-label overdosing regimen, as compared to within-range group.

**TABLE 2:** Basic characteristics of participants having within or out-of-expected-range edoxaban peak concentrations.

Characteristic	Peak (n = 76, 4 peak samples were missing)		
	Within range n = 49	Lower than range n = 6	Higher than range n = 21
Age (years)	74.9 ± 10.2	76.2 ± 7.2	71.6 ± 8.7
Male	32 (65.3)	4 (66.7)	11 (52.4)
Weight (kg)	65.1 ± 11.8	60.7 ± 6.8	67.8 ± 10.4
BMI (kg/m^2^)	25.7 ± 3.0	23.5 ± 1.6	25.4 ± 2.9
CRE (mg/dl)	1.2 ± 0.6	0.9 ± 0.1	1.0 ± 0.2
CrCL	52.7 ± 20.2	58.0 ± 13.5	60.7 ± 14.3
ALT (U/L)	21.7 ± 14.9	20.3 ± 9.0	29.4 ± 40.3
Comorbidities			
IS or TIA	26 (53.1)	3 (50.0)	9 (42.9)
CHF	8 (16.3)	0 (0)	3 (14.3)
Hypertension	34 (69.4)	6 (100.0)	13 (61.9)
Diabetes	7 (14.3)	2 (33.3)	5 (23.8)
MI or PAOD	6 (12.2)	0 (0)	3 (14.3)
Malignancy	6 (12.2)	2 (33.3)	6 (28.6)
Dyslipidemia	28 (57.1)	3 (50.0)	8 (38.1)
Bleeding history	15 (30.6)	2 (33.3)	8 (38.1)
ICH	3 (6.1)	0 (0)	2 (9.5)
GI bleeding	3 (6.1)	0 (0)	2 (9.5)
Other bleeding	10 (20.4)	2 (33.3)	4 (19.0)
CHADS2-VASc	3.9 ± 1.5	4.2 ± 1.5	3.8 ± 1.9
HAS-BLED	2.3 ± 1.0	2.7 ± 0.5	2.2 ± 1.3
Edoxaban concentration (ng/ml)			
Trough	22.3 ± 16.6	14.6 ± 15.4	28.8 ± 22.8
Peak^a^ ^,^ ^b^ ^,^ ^c^	199.5 ± 49.9	68.1 ± 25.1	382.6 ± 76.4
Edoxaban use			
60 mg daily^a^ ^,^ ^c^	14 (28.6)	3 (50.0)	19 (90.5)
30 mg daily	35 (71.4)	3 (50.0)	2 (9.5)
Off-label overdosing^a^ ^,^ ^c^	6 (12.2)	0 (0)	8 (38.1)
Off-label underdosing	6 (12.2)	2 (33.3)	1 (4.8)
Poor adherence	6 (12.5)	0 (0)	5 (23.8)
Concurrent medications^d^			
Amiodarone	11 (22.4)	2 (33.3)	3 (14.3)
Dronedarone	2 (4.1)	0 (0)	0 (0)
Verapamil	0 (0)	0 (0)	1 (4.8)
NSAID	2 (4.1)	0 (0)	1 (4.8)
Antiplatelet agents	3 (6.1)	0 (0)	1 (4.8)

The data are presented as numbers (proportions) or the means ± standard deviations. The 80 participants contributed 76 peak samples. ALT, alanine aminotransferase; BMI, body mass index; CHF, congestive heart failure; CrCL, creatinine clearance; CRE, serum creatinine; GI, gastrointestinal; ICH, intracranial hemorrhage; IS, ischemic stroke; MI, myocardial infarction; N/A, not applicable; NSAID, nonsteroidal anti-inflammatory drug; PAOD, peripheral arterial vascular disease; P-gp, p-glycoprotein; TIA, transient ischemic attack.

a Indicated differences between 3 groups.

b Indicated differences between the low peak and within-range peak groups.

c Indicated differences between the high peak and within-range peak groups.

d Concurrent medications: None of our patients concomitantly used quinidine, azole antifungal agents, protease inhibitors (P-glycoprotein inhibitors), rifampin, or enzyme-inducing antiepileptic drugs such as phenytoin and phenobarbital (P-glycoprotein inducers)

### Edoxaban Adherence

Twelve participants (15.6%) had poor edoxaban adherence, included 9 participants (18.8%) had within range trough concentration, 2 participants (10.5%) had lower- and 1 participant (11.1%) had higher-than-expected-range trough concentration. The difference was not significant, as listed in Table 1. The average trough concentration in the poor adherence group was lower than the good adherence group (22.7 ± 16.9 and 25.6 ± 23.0 ng/ml), but the difference was not significant (*p* = 0.90). The proportion of participants with poor adherence with lower-, within-, or higher-than-expected range peak concentration was not significantly different. And the average peak concentration was similar between poor and good adherence groups (263.5 ± 113.7 and 239.5 ± 110.4 ng/ml, *p* = 0.57).

### Appropriateness of the Edoxaban Prescription

**FIGURE 2:**
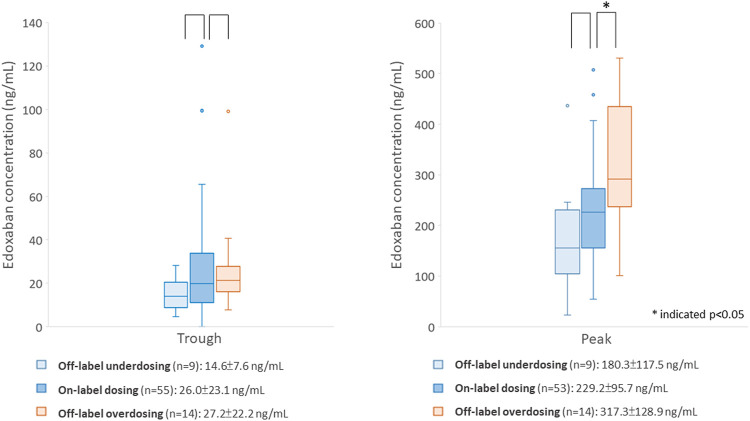
Comparison of edoxaban concentrations between participants using the on- or off-label dosing regimen.

The comparison between participants prescribed the off-label and on-label dosing regimens is listed in Supplementary Table S1. A total of 14 (17.5%) participants were ordered off-label overdosing regimens, and 10 (12.5%) participants were prescribed an off-label underdosing regimen. Among participants with overdosing regimen, 50.0% weighed ≤60 kg, and 57.1% had a CrCL< 50 ml/min. The peak concentration and proportion of participants with higher-than-expected range peak concentration were significantly different between 3 groups. Specifically, participants using the off-label overdosing regimen had significantly higher peak concentrations (317.3 ± 128.9 for the overdosing regimen versus 229.2 ± 95.7 ng/ml for the on-label dosing regimen, *p* = 0.01) and were more likely to have higher-than-expected-range peak concentrations (57.1% for the overdosing regimen versus 22.6% in the on-label dosing regimen, *p* = 0.02) than those with on-label dosing regimen. Nevertheless, the trough concentration was not significantly different between the three groups. Figure 2 depicts the differences in the edoxaban concentrations between the on-label and off-label dose groups. In addition, the proportion of cancer patients was different between 3 dosing groups. Specifically, participants prescribed an off-label underdosing regimen were more likely to have cancer history, compared with the participants taking the on-label dosing regimen (50.0 versus 16.1%).

### Factors Associated With Out-Of-Expected-Range Edoxaban Concentrations

The results of multivariate logistic regression of out-of-expected-range edoxaban concentration are listed in Table 3. To predict the lower-than-expected-range trough concentration, the association between increased CrCL and use of the 30 mg regimen was significant (odds ratio [OR] and 95% confidence interval [CI] were 1.06 [1.01, 1.11], *p* = 0.01 for CrCL and 5.77 [1.34, 24.75], *p* = 0.02 for 30 mg regimen). To predict higher-than-expected-range peak concentrations, the impact of using an off-label overdose regimen was significant (OR = 4.68 [1.24, 17.70], *p* = 0.02).

**TABLE 3:** Multivariate regression analysis of out-of-expected range edoxaban concentrations.

Trough lower than expected range		
**Factors**	**OR and 95% CI**	***p*** **-value**
Age (years)	1.03 (0.95, 1.11)	0.47
Male sex	0.34 (0.07, 1.61)	0.18
Weight (kg)	1.00 (0.93, 1.08)	1.00
CrCL (ml/min)	1.06 (1.01, 1.11)	**0.01**
Edoxaban dose (30 mg daily)	5.77 (1.34, 24.75)	**0.02**
**Peak higher than expected range**		
**Factors**	**OR and 95% CI**	***p*** **-value**
Age (years)	0.99 (0.93, 1.07)	0.85
Male sex	0.40 (0.09, 1.81)	0.23
Weight (kg)	1.05 (0.98, 1.13)	0.14
CrCL (ml/min)	1.01 (0.97, 1.05)	0.64
On-label regimen	References	--
Off-label underdosing regimen	0.32 (0.03, 3.12)	0.33
Off-label overdosing regimen	4.68 (1.24, 17.70)	**0.02**

CrCL, creatinine clearance, estimated using the Cockcroft-Gault formula; OR, odds ratio; 95% CI, 95% confidence interval.

Bold values indicated a p-value < 0.05.

In addition, we also examined the characteristics of participants with higher-than-expected-range trough concentrations and investigated factors associated with high trough levels, as the information was clinically important to reflect accumulation. To predict higher-than-expected-range edoxaban trough concentration, body mass index (BMI) was the only significant factor (OR = 0.54 [0.34, 0.87], *p* = 0.01).

### Participants With Fragile Characteristics

We further stratified all the participants into the fragile and nonfragile groups according to the criteria used in the ELDERCARE-AF trial. The comparisons between the two groups are listed in Table 4. Eight participants (10.0%) met the definition of fragile characteristics. These participants were older, had worse renal function, as reflected by lower CrCL, were more likely to have bleeding or intracerebral hemorrhage history, and had higher CHADS_2_-VASc and HAS-BLED scores than those in the nonfragile group. Fragile participants had higher trough concentration than non-fragile group (37.0 ± 31.7 vs. 23.7 ± 20.6 ng/ml), but the difference was not significant (*p* = 0.23). Similarly, the difference for peak concentration between fragile and non-fragile participants was not significant (fragile versus non-fragile, 231.6 ± 105.6 vs. 240.5 ± 2112.2 ng/ml, *p* = 0.69). None of the participants in fragile group had lower-than-expected range trough or peak concentrations, in contrast to 28.2% of trough concentrations and 8.7% of peak concentrations in fragile group were lower than the expected range. But the differences were non-significant.

**TABLE 4:** Comparisons between fragile and nonfragile participants.

Characteristic	Fragile n = 8	Nonfragile n = 72	*p*-Value
Age (years)	85.5 ± 3.6	73.2 ± 9.4	**<0.001**
Male	5 (62.5)	44 (61.1)	1.00
Weight (kg)	57.5 ± 13.6	65.8 ± 11.1	0.11
Weight ≤45 kg	2 (25.0)	3 (4.2)	0.08
BMI (kg/m^2^)	23.7 ± 4.0	25.0 ± 3.0	0.66
CRE (mg/dl)	1.1 ± 0.3	1.1 ± 0.5	0.48
CrCL	40.6 ± 17.4	56.6 ± 18.1	**0.02**
CrCL 15–30 ml/min	2 (25.0)	2 (2.8)	0.05
ALT (U/L)	18.6 ± 12.0	24.8 ± 25.0	0.24
CHADS_2_-VASc	5.1 ± 1.7	3.7 ± 1.6	**0.04**
HAS-BLED	3.5 ± 0.5	2.1 ± 1.1	**0.001**
Comorbidities			
IS or TIA	5 (62.5)	35 (48.6)	0.71
CHF	2 (25.0)	11 (15.3)	0.61
Hypertension	7 (87.5)	47 (65.3)	0.26
Diabetes	2 (25.0)	12 (16.7)	0.62
MI or PAOD	1 (12.5)	9 (12.5)	1.00
Malignancy	1 (12.5)	14 (19.4)	1.00
Dyslipidemia	4 (50.0)	36 (50.0)	1.00
Bleeding history	7 (87.5)	20 (27.8)	**0.002**
ICH	5 (62.5)	1 (1.4)	**<0.001**
GI bleeding	1 (12.5)	4 (5.6)	0.42
Other bleeding	1 (12.5)	16 (22.2)	1.00
Edoxaban level (ng/ml)			
Trough	37.0 ± 31.7	23.7 ± 20.6	0.23
Higher than expected	2 (28.6)	7 (9.7)	0.18
Lower than expected	0 (0)	20 (28.2)	0.18
Peak	231.6 ± 105.6	240.5 ± 112.2	0.69
Higher than expected	1 (14.3)	20 (29.0)	0.67
Lower than expected	0 (0)	6 (8.7)	1.00
Edoxaban use			
30 mg daily	7 (87.5)	36 (50.0)	0.06
Concurrent use of NSAIDs or antiplatelet agents^c^	1 (12.5)	5 (6.9)	0.48

The data are presented as numbers (proportions) or the means ± standard deviations. The 80 participants contributed 78 trough samples and 76 peak samples. The trough concentrations were not available in 1 fragile participant and 1 nonfragile participant. The peak concentrations were not available in 1 fragile participant and 3 nonfragile participants. ALT, alanine aminotransferase; BMI, body mass index; CHF, congestive heart failure; CrCL, creatinine clearance; CRE, serum creatinine; ICH, intracranial hemorrhage; IS, ischemic stroke; MI, myocardial infarction; NSAID, nonsteroidal anti-inflammatory drug; PAOD, peripheral arterial vascular disease; P-gp, p-glycoprotein; TIA, transient ischemic attack.

Bold values indicated a p-value < 0.05.

## Discussion

This study reported real-world data on edoxaban concentrations in patients with AF. Our data showed that approximately one-fourth of the trough edoxaban concentrations were lower than the expected range reported in clinical trials, and approximately one-fourth of the peak edoxaban concentrations were higher than the expected range. A low trough concentration was associated with good renal function and the use of the 30 mg regimen, while a high peak concentration was associated with an off-label overdosing regimen.

Edoxaban concentration has been reported in some Asian cohorts, including the subanalysis of the ENGAGE AF-TIMI 48 trial in Asian patients and the Cardiovascular Institute Academic Research Organization (CVI ARO 7) cohort in Japan, which enrolled AF patients (Chao et al., 2019; Suzuki et al., 2020). Although our present cohort enrolled older participants with lower CrCL and higher CHADS2 or CHA_2_DS_2_-VASc scores compared to the patients in those two cohorts, the reported edoxaban concentrations were not significantly different between cohorts (median [IQR], ENGAGE-TIMI 48: 25 [13.9–47.4] and ours: 19.6 [11.8–30.1] ng/ml; median [5th-95th percentile], CVI ARO 7: 14.4 [4.8–40.7] and ours 19.6 [4.8–65.6] ng/ml) (Chao et al., 2019; Suzuki et al., 2020). However, these investigations did not examine factors associated with high or low edoxaban concentrations.

Our data showed that the edoxaban concentration was influenced by the dose regimen. Use of the 30 mg regimen was associated with lower-than-expected-range trough concentrations. To further address whether the edoxaban regimen was appropriate, the results showed that compared to participants prescribed an on-label dosing regimen, those prescribed an off-label overdosing regimens had significantly increased peak concentrations. The dose adjustment criteria for edoxaban are considered to be complicated. Among the participants prescribed off-label dosing regimens, approximately 60% received higher than the recommended dose. More than half of the patients who received the overdose regimen received this regimen due to nonadherence to the CrCL criteria for dose reduction. The association between the appropriateness of the NOAC regimen and clinical outcomes was investigated in a cohort study in Taiwan. The off-label overdose regimen increased the risk of major bleeding compared to the on-label regimen (Chan et al., 2020). Our data provide pharmacological evidence to support this finding. Taken together, physicians should be aware of the importance of prescribing edoxaban doses according to the label. Implementation of a clinical decision support system or order verification by the clinical pharmacist may improve the accuracy of prescription.

Our results indicated that the edoxaban concentration was also influenced by patient characteristics. A higher CrCL predicted a lower-than-expected range trough concentration. In facts, the renal clearance of unchanged drug accounted for 50% of the total clearance of edoxaban (Parasrampuria and Truitt, 2016). Exposure to edoxaban is decreased in patients with renal impairments compared with those with normal renal function (Bohula et al., 2016; Parasrampuria and Truitt, 2016). Our data were consistent with the pharmacokinetic properties of edoxaban. We also found that a lower BMI was associated with increased risk of higher-than-expected-range trough concentration. In the ENGAGE-TIMI 48 trial, edoxaban concentration was similar across BMI groups ≥18.5 kg/m^2^ (Boriani et al., 2019). The causes for our inconsistent finding were multifactorial. First, our cohort was generally thinner. Around 60% of our participants had normal BMI (i.e., 18.5 to <25 kg/m^2^) and 40% of the participants had overweight BMI (25–30 kg/m^2^), in comparison to 21.4% of patients with normal BMI and 37.6% patients with overweight BMI in the ENGAGE-TIMI 48 trial (Boriani et al., 2019). Second, 22% of participants with low bodyweight (≤60 kg) did not received reduced edoxaban dose regimen. Asian population were thinner than the Western populations in general, whether the impact of BMI on edoxaban exposure is clinically significant remained unclear from our data.

The results of the ELDERCARE-AF trial showed that the administration of a very low-dose edoxaban regimen (i.e., 15 mg daily) to very elderly AF patients with fragile characteristics still reduced the risk of stroke without increasing the risk of bleeding compared with the administration of no treatment (Okumura et al., 2020). However, the edoxaban concentration was not reported. We analyzed the effect of fragility characteristics on the edoxaban concentration and did not find a difference between groups. Interestingly, none of the participants in the fragile group had lower-than-expected edoxaban concentrations, either for the trough or for the peak concentrations. Due to the small participant size in the fragile group and the nonsignificant differences between the two groups, we were not able to conclude that adjusting the dose of edoxaban from 30 mg to a very-low-dose regimen of 15 mg daily is appropriate from the perspective of pharmacological activity. Future large-scale studies that include the outcomes are necessary to answer this question.

The association between the edoxaban concentration and clinical outcome was also reported in the ENGAGE-TIMI 48 subanalysis. In Asian populations with edoxaban trough concentrations less than 15 ng/ml, the risk of ischemic stroke exceeds that of major bleeding. Moreover, when edoxaban trough concentrations are greater than 90 ng/ml, the risk of ICH exceeds that of ischemic stroke (Chao et al., 2019). If we apply this range, 28 participants (35.9%) had edoxaban concentrations lower than the concentration attributed to an increased risk of ischemic stroke. In contrast, only 3 participants (3.9%) had edoxaban concentrations higher than the concentration attributed to an increased risk of ICH. Further investigation is required to determine whether a low concentration is associated with worse outcomes in the real world. Importantly, our study highlights the significance of measuring the edoxaban concentration, especially in patients who are at risk to changes in edoxaban exposure, such as patients with good renal function, low BMI and fragility.

We acknowledge several limitations of our study. First, this observational study was conducted in a single center, and our results may not be extrapolated to other health care facilities due to differences in patient characteristics. Second, we did not provide pharmacokinetic parameters to support our observation. Nevertheless, only two blood samples were collected during the dosing interval, and thus, the pharmacokinetic parameters of our data may not be accurate. In addition, the dosing regimen for edoxaban is relatively fixed, and the use of pharmacokinetic parameters to precisely determine the dose may not considerably change clinical practice. Third, this is an observational study, and the time of peak and trough sampling varied within a range, which may cause changes in blood concentrations. Last, the sample size was small, and we did not incorporate the outcome analysis. Larger-scale studies with a longer follow-up duration are required to determine whether these observations were linked to worse outcomes.

In conclusion, this study provides the first real-world data on the edoxaban concentration in patients with AF. A low edoxaban concentration was associated with better renal function and the use of a 30 mg regimen, while a high edoxaban concentration was related to an off-label overdosing regimen. Physicians should be familiar with the criteria for adjusting the dose of edoxaban and avoid off-label dosing.

## Data Availability

The raw data supporting the conclusions of this article will be made available by the authors, without undue reservation.
