# Supplementary material for: Factors Associated With Edoxaban Concentration Among Patients With Atrial Fibrillation
Source: Front Pharmacol. 2021 Sep 9;12:736826. doi: 10.3389/fphar.2021.736826 (PMC8458830; doi:10.3389/fphar.2021.736826)
Supplement: Supplementary file 1 [file Table1.DOCX]

Supplementary Material

# Measurement of the plasma edoxaban concentration:

Stock solutions of edoxaban and [*d*_6_]-edoxaban were each prepared at a concentration of 1.0 mg mL^-1^ in methanol. The working solutions of edoxaban were prepared at a concentration of 100 µg mL^-1^ by diluting the stock solution with methanol. All solutions were stored at -20°C.

The plasma edoxaban concentration was measured immediately before edoxaban administration (trough) and 1 to 4 hours after edoxaban administration (peak) by collected blood in tubes containing K2EDTA (BD Vacutainer®). Blood samples were centrifuged using a standard procedure to obtain plasma and then stored in a -80°C freezer. For sample extraction, 100 μL of plasma were extracted with 800 μL of methanol by shaking for 2 min at 1000 rpm using a Geno/Grinder 2010 (SPEX® Sample Prep, Metuchen, NJ). The extract was then centrifuged at 15,000 rcf for 5 minutes. Then, 400 μL of supernatant were transferred to a new Eppendorf tube. The plasma extracts were dried in a centrifugal vaporizer (Thermo SpeedVac® SPD111V, Waltham, MA, US). The residue was reconstituted with 200 μL of methanol followed by Geno/Grinder mixing at 1000 rpm for 3 min and then centrifuged at 15,000 rcf for 5 min. The supernatant was filtered through a 0.2-μm PP membrane filter (RC-4, Sartorius, Göttingen, Germany) and then analyzed using ultrahigh-performance liquid chromatography with tandem mass spectrometry (UHPLC-MS/MS).

LC separations were performed using an Agilent 1290 UHPLC system equipped with a binary solvent pump, an autosampler, a sample reservoir, and a column oven (Agilent Technologies, Waldbronn, Germany). The coupled mass spectrometer was an Agilent 6460 triple quadrupole system (Agilent Technologies, Waldbronn, Germany). A Kinetex reverse-phase core-shell C18 column (2.1 × 50 mm, 2.6 μm, 100 Å, Phenomenex, Torrance, CA, USA) was used for separation. The mobile phase consisted of 0.1% formic acid and 10 mM ammonium acetate in water (solvent A), and 0.1% formic acid and 10 mM ammonium acetate in isopropanol and ACN (9:1, v/v) (solvent B). The flow rate was 0.35 mL min^-1^. The gradient profile started with 0% B for 0.5 min, then changed to 6% B in 0.1 min and remained at 6% B for 0.6 min, subsequently increased to 25% B in 0.5 min, 27.5% B in 0.5 min, and 50% B in 0.5 min, and was maintained 50% B for 1 min. Finally, the column was re-equilibrated to 0% B for 2 min until the next injection. The temperature of the sample reservoir was maintained at 4°C, and the column oven temperature was set to 55°C. The injection volume was 3 μL.

Positive electrospray ionization mode was utilized with the following parameters: 350°C drying gas temperature, 10 L min^-1^ drying gas flow rate, 45 psi nebulizer pressure, 350°C sheath gas temperature, 11 L min^-1^ sheath gas flow rate, 3500 V capillary voltage, and 500 V nozzle voltage. MS acquisition was executed using multiple reaction monitoring (MRM) mode. Two transitions were selected as a quantifier and qualifier: 548.1🡪 366.1 and 548.1 🡪 152 for edoxaban and 554.1 🡪 372.1 and 554.1 🡪 158.1 for [*d*_6_]-edoxaban, respectively.

The validation data showed that the coefficient of determination was greater than 0.999 within the range of 1 to 750 ng mL^-1^. Precision (Intra-assay variability) and accuracy were evaluated at three concentration levels (5 ng/mL, 50 ng/mL and 500 ng/mL). Precision was within relative standard deviation of 5%, and the accuracy was within 100±10%. The carry-over effect was evaluated by comparing peak area of the highest QC sample (500 ng/mL) and that of a subsequent blank sample. The carry-over was less than 20% of lowest concentration of the linear range. LOD and LOQ were 0.3 ng/mL and 0.1 ng/mL, respectively, which were calculated using the followed equations.

$$LOQ=\frac{10\sigma}{S}$$

$$LOD=\frac{3.3\sigma}{S}$$

Where σ is the standard deviation of the lowest concentration ratio from the calibration curve and S is the slope of the calibration curve.

Table S1. Comparison between participants treated with on- or off-label edoxaban dosing regimens.

| Characteristic | On-label regimen  n=56 | Off-label underdosing regimen  (30 mg daily)  n=10 | Off-label overdosing regimen  (60 mg daily)  n=14 |
| --- | --- | --- | --- |
| Age (years) | 74.2±9.5 | 77.5±11.1 | 72.8±10.0 |
| Male | 37 (66.1) | 6 (60.0) | 6 (42.9) |
| Weight (kg) | 64.8±12.9 | 67.9±5.5 | 63.3±9.0 |
| BMI (kg/m^2^) | 24.3±3.4 | 25.4±2.3 | 25.0±2.3 |
| CRE (mg/dL) | 1.2±0.5 | 0.9±0.2 | 1.0±0.3 |
| CrCL | 53.9±20.7 | 60.6±7.6 | 55.5±14.5 |
| Comorbidities |  |  |  |
| IS or TIA | 31 (55.4) | 2 (20.0) | 7 (50.0) |
| CHF | 9 (16.1) | 2 (20.0) | 2 (14.3) |
| Hypertension | 39 (69.6) | 6 (60.0) | 9 (64.3) |
| Diabetes | 10 (17.9) | 1 (10.0) | 3 (21.4) |
| MI or PAOD | 8 (14.3) | 0 (0) | 2 (14.3) |
| Malignancy^*,†^ | 9 (16.1) | 5 (50.0) | 1 (7.1) |
| Dyslipidemia | 33 (58.9) | 3 (30.0) | 4 (28.6) |
| Bleeding history | 22 (39.3) | 3 (30.0) | 2 (14.3) |
| ICH | 6 (10.7) | 0 (0) | 0 (0) |
| GI bleeding | 4 (7.1) | 1 (10.0) | 0 (0) |
| Other bleeding | 13 (23.2) | 2 (20.0) | 2 (14.3) |
| CHADS_2_-VASc | 3.9±1.6 | 3.2±1.1 | 4.1±1.9 |
| HAS-BLED | 2.5±1.1 | 1.8±1.0 | 1.9±1.1 |
| Edoxaban concentration (ng/mL) |  |  |  |
| Trough | 26.0±23.1 | 14.6±7.6 | 27.2±22.2 |
| Higher than expected | 8 (14.5) | 0 (0) | 1 (7.1) |
| Lower than expected | 14 (25.5) | 4 (44.4) | 2 (14.3) |
| Peak^*,‡^ | 229.2±95.7 | 180.8±117.5 | 317.3±128.9 |
| Higher than expected^*,‡^ | 12 (22.6) | 1 (11.1) | 8 (57.1) |
| Lower than expected | 4 (7.5) | 2 (22.2) | 0 (0) |
| Dose adjustment criteria |  |  |  |
| Weight ≤ 60 kg^*,†^ | 19 (33.9) | 0 (0) | 7 (50.0) |
| CrCL < 50 mL/min^*,†^ | 28 (50.0) | 0 (0) | 8 (57.1) |
| P-gp inhibitor use^§^ | 2 (3.6) | 0 (0) | 0 (0) |
| Abbreviations: BMI, body mass index; CHF, congestive heart failure; CrCL, creatinine clearance; CRE, serum creatinine; GI, gastrointestinal; ICH, intracranial hemorrhage; IS, ischemic stroke; MI, myocardial infarction; PAOD, peripheral arterial vascular disease; P-gp, p-glycoprotein; TIA, transient ischemic attack.  Data are presented as numbers (proportions) or means ± standard deviations. The 80 participants contributed 78 trough concentrations and 76 peak concentrations. The trough concentration was not available for 1 participant receiving the off-label underdosing regimen and 1 participant receiving the on-label dosing regimen. The peak concentration was not available for 1 participant receiving the off-label overdosing regimen and 3 participants receiving the on-label dosing regimen.  ^*^indicated differences between 3 groups.  ^†^indicated differences between the off-label underdosing regimen and on-label regimen.  ^‡^indicated differences between the off-label overdosing regimen and on-label regimen.]  ^§^P-glycoprotein inhibitors include dronedarone, cyclosporin, erythromycin, and ketoconazole. | | | |
